# Validity and reliability of Gripwise digital dynamometer in the assessment of handgrip strength in older adults

**DOI:** 10.3389/fragi.2025.1560097

**Published:** 2025-04-25

**Authors:** Gabriela Benatti de Oliveira, Lara Vilar Fernandes, Teresa F. Amaral, Ana Carolina Junqueira Vasques, Ligiana Pires Corona

**Affiliations:** ^1^ Faculty of Medical Sciences, State University of Campinas, Campinas, São Paulo, Brazil; ^2^ Faculty of Applied Sciences, State University of Campinas, Limeira, São Paulo, Brazil; ^3^ Faculty of Nutrition and Food Sciences UPorto, University of Porto, Porto, Portugal

**Keywords:** handgrip strength, dynamometer, dynapenia, aging, reliability

## Abstract

**Introduction:**

With the advancement of studies on the importance of sarcopenia in the aging process, new technologies have been developed to assess muscle mass and function. However, most research on portable devices has not considered a wide range of ages and clinical conditions. This study aimed to evaluate the reliability of the Gripwise digital dynamometer in measuring handgrip strength in older Brazilian adults, comparing its performance with the widely used Saehan device.

**Methods:**

A cross-sectional study was conducted with 149 participants (32 men and 117 women), with an average age of 69.5 years. Handgrip strength was measured using both the Gripwise and Saehan dynamometers. Reliability was assessed using the intraclass correlation coefficient (ICC). Analyses considered three handgrip strength measurements from both devices, as well as the highest value obtained. The classification of dynapenia (low muscle strength) was compared using different cutoff points proposed by Villain et al. (2023), Spexoto et al. (2022), and Cruz-Jentoft et al. (2019).

**Results:**

Both dynamometers demonstrated excellent reliability, with ICC values above 0.90. However, significant differences in mean handgrip strength values were observed between the devices (approximately 3.5–four kgf). These variations impacted the classification of dynapenia, with the Gripwise identifying more cases of low muscle strength compared to Saehan.

**Conclusion:**

The lower values reported by the Gripwise may impact clinical decision-making in two ways. On one hand, lower values may lead to earlier detection of muscle weakness, allowing for quicker intervention in individuals with strength below typical thresholds. However, this could also result in an overestimation of the prevalence of dynapenia if the values do not accurately reflect true muscle strength, which could lead to unnecessary interventions. Therefore, it is crucial to consider the need for adjustments in the cutoff points when using Gripwise. These findings highlight the need to revise cutoff points for dynapenia classification, considering device variations and model differences in older age groups.

## Introduction

With the increase in life expectancy, there is a significant rise in the prevalence of older adults with excess body fat and/or reduced muscle function and mass, often resulting in conditions such as sarcopenia and sarcopenic obesity (SO) (Benz et al., 2024). These conditions are associated with increased morbidity and mortality, and their effective detection and treatment remain limited in clinical practice (Benz et al., 2024). Handgrip strength is widely used to assess muscle function and physical competence, playing a crucial role in maintaining the strength needed for daily activities during aging (Chen et al., 2023). Besides being an important biomarker for overall muscle function, handgrip strength is also associated with slower epigenetic aging, suggesting a protective effect. Studies indicate that handgrip strength is a relevant indicator of vitality and is strongly associated with successful aging, being crucial for evaluating the ability to cope with the challenges of aging (Zhao et al., 2023; Lu et al., 2023). Furthermore, a study demonstrated a significant correlation between handgrip strength and aging-related laboratory parameters, including leukocytes, absolute neutrophils, absolute lymphocytes, NLR, and ESR. These findings reinforce the notion that handgrip strength may serve as a simple and effective predictor of health status in older adults, reflecting biological changes associated with aging (Kemala Sari et al., 2025).

In contrast, another study found that handgrip strength was only moderately to weakly associated with functional outcomes in older women, suggesting that it may not fully capture overall functional capacity. This indicates that handgrip strength alone may not be sufficient to generalize conclusions about global functionality (Muollo et al., 2025).

Technological advancements have significantly improved dynamometry, particularly with the shift from analogic to digital dynamometers (Silva-Santos et al., 2024). While analogic dynamometers provide objective measurements of isometric maximum strength, they can be limited by variables such as the type of dynamometer, measurement protocol, and hand position, which affect result accuracy. In contrast, digital dynamometers offer a more detailed analysis of muscle function, enabling a more comprehensive assessment through force-time curves (Silva-Santos et al., 2024). These devices are appealing due to their portability, ease of use, and lower cost compared to isokinetic devices. Among the evaluated devices, the Gripwise stands out for its characteristics, featuring a reduced weight (290 g), which may be advantageous in the assessment of individuals with malnutrition, frailty, and advanced age (Karagiannopoulos et al., 2022).

Assessing handgrip strength in older adults presents specific challenges, including significant variations with age and sex. For instance, women aged 60 to 69 have an average grip strength of 21.7 ± 5.5 kg, whereas men in the same age group have an average of 32.9 ± 8.7 kg. These differences increase with age, and handgrip strength is strongly correlated with appendicular skeletal muscle mass and nutritional status (Soraya and Parwanto, 2023). Several factors, such as hand dominance, body mass, and psychological influences, contribute to variations in handgrip strength, emphasizing the need for standardized measurement protocols to ensure comparability across populations (Quattrocchi et al., 2024). In this context, the largest global study on handgrip strength, encompassing 2.4 million adults worldwide, provides age- and sex-specific norms. It shows that, on average, handgrip strength improves slightly during early adulthood, peaks between 30 and 39 years (49.7 kg for males and 29.7 kg for females), then declines with age. This age-related decline accelerates from middle to late adulthood, with a slightly greater decrease in males than in females during middle age (Tomkinson et al., 2025).

The validity and reliability of measurement devices, such as the Gripwise dynamometer, have been evaluated against the Jamar hydraulic, a gold standard in dynamometry. Recent studies show that while Gripwise is reliable and offers ergonomic advantages, there is a need to validate alternative cutoffs and consider handle ergonomics to improve handgrip strength measurement reliability (Villain et al., 2023; Guerra et al., 2017).

However, most studies on portable equipment do not cover a wide range of ages and clinical conditions. This study aims to assess the validity and reliability of the Gripwise digital dynamometer in older Brazilian adults, contributing to the analysis of this instrument and promoting early diagnosis and interventions to improve nutritional status and quality of life for older adults over time.

## Materials and methods

### Design and subjects

This quantitative, cross-sectional study analyzed data from 149 older adults (aged 60 and over) living in Campinas, Brazil. Participants were recruited from three sources: 1) individuals taking part in the UniversIDADE Program at the State University of Campinas (UNICAMP) (Riedo et al., 2017; State University of Campinas UNICAMP, 2017), which provides workshops and activities for people in pre-retirement, retirement, and post-retirement; 2) UNICAMP employees, and 3) patients attending the Geriatric Outpatient Clinic at the Clinics Hospital of UNICAMP. The study was approved by the Ethics Committee at UNICAMP (Approval Number: 51443321.0.0000.5404). All participants signed an Informed Consent Form.

To be included in the study, participants had to be at least 60 years old, live in Campinas or the surrounding areas, have suitable neurological and cognitive health to complete questionnaires and present adequate mobility for physical assessments. Exclusion criteria were receiving home care or undergoing chemotherapy, presence of conditions that significantly alter body composition (e.g., chronic obstructive pulmonary disease (COPD), chronic kidney disease requiring dialysis, Parkinson’s disease, congestive heart failure), or HIV positive. More details about the recruitment and the sample have been published previously (Benatti de Oliveira et al., 2024; Fernandes et al., 2024).

### Handgrip strenght

The GRIPWISE^®^ dynamometer (Gripwise Tech P, 2023) (GripWise Tech, Portugal) and the SAEHAN^®^ dynamometer (Corporation, 2020) (Saehan Corporation–SH5001, Korea) were used to measure each participant’s grip strength. Both dynamometers were calibrated before data collection began. The calibration of the digital dynamometer included a visual inspection, device stabilization, zeroing verification, and pairing with the mobile software. The calibration of the analog dynamometer involved inspecting the pointers and ensuring they were in the zero position before use. The Saehan analog dynamometer, with dimensions of 29 cm × 20 cm × 11 cm and weighing 1.4 kg, has a resolution of two kgf. It was validated using the Jamar dynamometer as a reference in an adult population, with 100 healthy individuals aged between 20 and 50 years old (Reis and Arantes, 2011).

The commercial version of BodyGrip, called Gripwise, connects to iOS and Android apps, a web app, and a cloud platform, with data stored on two European servers. It was verified using a Griptester, which employs a custom load cell and a mobile app to collect data and ensure system calibration. The app offers automatic, semi-automatic, and manual calibration modes, with results recorded remotely for online consultation (Restivo et al., 2023). The equipment is lightweight (290 g), with compact dimensions of 15 cm × 4.4 cm x 3.5 cm, and a resolution of 100 gf (Gripwise Tech P, 2023).

Participants were comfortably seated in an armless chair, with their feet on the floor and their hips and knees flexed at approximately 90°. The tested arm was positioned with the shoulder adducted and in neutral rotation, the elbow flexed at 90°, the forearm in a neutral position, and the wrist between 0 and 30 degrees of extension and 0–15 degrees of adduction. Participants were instructed to maintain this position during the tests and were adjusted by the examiner as needed (MacDermid et al., 2015). All accessories, such as watches, bracelets, rings, and bangles, were removed from both upper limbs before testing. Evaluations were conducted individually in a private setting by the same examiner. The data collection was conducted by two researchers, both postgraduate students who received proper training and standardization. During the handgrip strength measurement, verbal encouragement was provided to the participant in all three recorded measurements for both dynamometers, to exert maximum force. After proper positioning, three consecutive measurements were taken with the dominant hand using the first device and then with the second device, the order of dynamometer applicability was randomized. A 10 s interval between measurements and a 1 min rest between successive measurement sets were observed, following the recommendations of Watanabe et al. (2005).

### Statistical analyses

Reliability analyses were conducted considering the three handgrip strength measurements from the digital and analogic devices and the maximum handgrip strength value obtained. For the maximum handgrip strength analysis, descriptive statistics including mean, median, and standard deviation were computed. The Wilcoxon signed-rank test was also performed to assess whether there was a statistically significant difference in maximum grip strength between the two devices.

Intra-rater reliability was assessed using intraclass correlation coefficients (ICC) with a 95% confidence interval, employing a two-way mixed model with absolute agreement, following the methodology of previous reliability studies (Chandler et al., 2020; Wagner et al., 2016). ICC values were classified as poor (<0.50), moderate (0.50–0.75), good (0.75–0.90), or excellent (>0.90) (Hallgren, 2012; Santiago-Nuño et al., 2019). Bland-Altman plots were used to assess individual differences (Martin Bland and Altman, 1986; Wing et al., 2021). Limits of Agreement (LOA) were analyzed to identify trends of homogeneity or heterogeneity and are often represented in Bland-Altman plots as ±1.96 standard deviations, indicating test-retest reliability for 95% of the confidential interval (McGirr et al., 2021). All analyses were conducted for the total sample and subsequently stratified by sex and age range, with three ranges (60–69 years, 70–79 years, and 80+ years).

The definitions of dynapenia were established and tested based on different cutoff points for handgrip strength. The cutoff by Villain et al. (2023), <12 kgf for women, and <22 kgf for men) was specifically established for the Gripwise digital dynamometer; Spexoto et al. (2022) (<23 kgf for women and <36 kgf for men) and Cruz-Jentoft et al. (2019) (<16 kgf for women and <27 kgf for men).

Sensitivity, specificity, Positive Predictive Value (PPV), and Negative Predictive Value (NPV) were calculated to determine the accuracy of handgrip strength in identifying dynapenia, using both dynamometers, with the Saehan serving as the comparison dynamometer. Dynapenia identification followed different cutoff points and dynamometers for comparison, and analyses were conducted in three distinct models, as follows:

• Model 1 = Comparison of low strength classification by Spexoto et al. (2022) (< 23 kgf for women and <36 kgf for men) using the Saehan dynamometer with low strength classification by Villain et al. (2023) (< 12 kgf for women and <22 kgf for men) using the Gripwise dynamometer.
• Model 2 = Comparison of low strength classification by Spexoto et al. (2022) (< 23 kgf for women and <36 kgf for men) using both the analogic and Gripwise dynamometers.
• Model 3 = Comparison of low strength classification by Cruz-Jentoft et al. (2019) (< 16 kgf for women and <27 kgf for men) using both the Saehan and Gripwise dynamometers.

To evaluate the agreement between diagnoses obtained with the Saehan (reference) and Gripwise devices, the kappa coefficient and its 95% confidence interval (CI) were calculated, followed by an asymptotic test for the simple kappa coefficient, with the null hypothesis set at kappa (*κ)* = 0. The level of agreement was interpreted according to Cohen’s kappa scale: *κ* between 0 and 0.20 indicates no agreement; 0.21–0.39, minimal agreement; 0.40–0.59, weak agreement; 0.60–0.79, moderate agreement; 0.80–0.90, strong agreement; and above 0.90, almost perfect agreement (McHugh, 2012). It is a statistic used to test the reliability between raters (interrater) or within the same rater (intrarater), quantifying the agreement and adjusting for the possibility of agreement occurring by chance (McHugh, 2012).

All analyses were performed using Stata 14 (College Station, Texas) software and Jasp (0.19.1) software, with a significance level of 5%.

## Results

We conducted a cross-sectional study with 149 participants (32 men and 117 women), with an average age of 69.5 years and an average BMI (Body Mass Index) of 28.8 kg/m^2^, with a minimum value of 18.5 kg/m^2^ and a maximum of 42.1 kg/m^2^.

### Comparison of measurements between both devices

The descriptive comparisons between the Saehan and Gripwise dynamometers are shown in Table 1. We observed a significant difference in the mean values between the dynamometers, both in the three individual measurements (approximately four kgf) and in the maximum value obtained (difference of 3.5 kgf). This significant difference refers to the variation between the individual mean values and the maximum values obtained from each dynamometer, which may lead to errors in classifying impairments in handgrip strength. According to the analysis using the Wilcoxon signed-rank test, this difference in medians was confirmed, with the Saehan dynamometer showing higher values compared to Gripwise (z = 9.737; p < 0.001). Regarding Table 1, the results show that the Saehan presents consistently higher standard deviations (SDs) in all measurements compared to the Gripwise. This indicates that the measurements taken with the Saehan have greater variability, either between individuals or across different attempts. In contrast, the Gripwise shows lower SD values, ranging from 7.43 to 7.63 kgf, i.e., a smaller data dispersion.

**TABLE 1:** Descriptive comparison between the Saehan and Gripwise dynamometers.

	Dynamometer					
	Saehan			Gripwise		
Measures	Mean (kgf)	SD	Min.-max. (kgf)	Mean (kgf)	SD	Min.-max. (kgf)
1	25.3	7.88	(10.00; 52.00)	21.55	7.47	(6.50; 49.10)
2	25.5	7.78	(12.00; 54.00)	22.41	7.62	(7.90; 50.90)
3	26.0	7.68	(12.00; 51.00)	21.95	7.43	(5.90; 48.30)
Maximum handgrip strength	26.8	7.81	(12.00; 54.00)	23.27	7.63	(8.00; 50.90)

Note: SD, standard deviation; Min = minimum; Max = maximum.

### Intra-rater reliability

Table 2 displays the reliability of the three measurements for each dynamometer evaluated. Most ICC values were considered excellent (>0.90), with the lowest values observed in the sample of women using the Saehan (0.88), which were still considered good.

**TABLE 2:** Intraclass Correlation Coefficient (ICC) for the three measurements obtained with Saehan and with the Gripwise.

Dynamometer	N	ICC	95% CI		*p*
Gripwise					
Total Sample	149	0.95	0.93	0.96	<0.001
Men	32	0.89	0.82	0.94	<0.001
Women	117	0.90	0.86	0.92	<0.001
Age Range					
60–69 years	74	0.96	0.94	0.97	<0.001
70–79 years	64	0.93	0.89	0.95	<0.001
80+ years	11	0.94	0.84	0.98	<0.001
Saehan					
Total Sample	149	0.95	0.94	0.96	<0.001
Men	32	0.90	0.83	0.95	<0.001
Women	117	0.88	0.84	0.91	<0.001
Age Range					
60–69 years	74	0.95	0.93	0.97	<0.001
70–79 years	64	0.94	0.91	0.96	<0.001
80+ years	11	0.95	0.88	0.99	<0.001

Note: ICC, intraclass correlation coefficient; 95% CI, 95% confidence interval.

**FIGURE 1:**
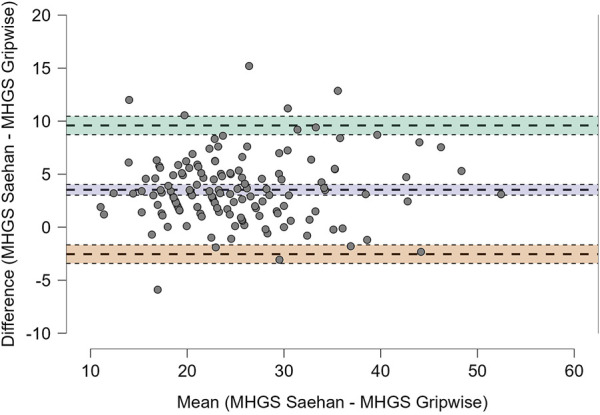
Bland-altman plot: Difference in maximum measured HandGrip strength (kgf) between dynamometers (saehan–gripwise).

In Table 3, we compare the maximum handgrip strength measured with the Saehan and Gripwise dynamometers. We found that the ICC values variation was wider, ranging from moderate to good, with the lowest values in the sample of women (0.65) and the age group of 80 years or older (0.69), both considered moderate. In Figure 1, we present the Bland-Altman plot of this comparison. Most of the bias between measures is observed around five kgf.

**TABLE 3:** Comparison of maximum handgrip strength measured with the Saehan and Gripwise and Intraclass Correlation Coefficient (ICC).

Maximum handgrip strength	ICC	95% CI		p	LOA
Gripwise x Saehan					
Total Sample	0.92	0.98	0.94	<0.001	3.52 (−2.55; 9.59)
Men	0.72	0.01	0.90	<0.001	4.37 (−2.77; 11.51)
Women	0.65	0.02	0.85	<0.001	3.29 (−2.40; 8.98)
Age Range					
60–69 years	0.86	0.37	0.95	<0.001	2.90 (−2,77; 8.57)
70–79 years	0.82	0.09	0.94	<0.001	3.99 (−2.18; 10.17)
80+ years	0.69	−0.08	0.93	<0.001	4.97 (-1.74; 11.69)

Note: ICC, intraclass correlation coefficient; 95% CI, 95% Confidence Interval; LOA, limits of agreement.

### Analyzes according to cutoff points

In Table 4, classifications of low handgrip strength (dynapenia) were performed according to each protocol and the dynamometer used. This allows us to compare how each device classified the participants differently under the same cutoff standard. In the cutoff point of Villain et al. (2023) using the reference dynamometer, all participants were classified as non-dynapenic. In contrast, according to the Gripwise values, seven were classified as dynapenic.

**TABLE 4:** Descriptive analyzes on the classification of maximum handgrip strength according to cutoff points in a sample of older Brazilian adults.

Cutoff points	Dynamometer			
	Saehan		Gripwise	
	Non dynapenic (n)	Dynapenic (n)	Non dynapenic (n)	Dynapenic (n)
Villain et al. (2023) (< 12 kgf for women and <22 kgf for men)	149	0	142	7
Spexoto et al. (2022) (< 23 kgf for women and <36 kgf for men)	90	59	45	104
Cruz-Jentoft et al. (2019) (< 16 kgf for women and <27 kgf for men)	144	5	125	24

In Table 5, we present analyses of agreement between the recommended cutoff points for low handgrip strength using the Saehan and Gripwise dynamometers according to the defined models.

**TABLE 5:** Agreement between the recommended cutoff points for low handgrip strength using the Saehan and Gripwise dynamometers in older Brazilian adults.

	Non-dynapenic	Dynapenic	Total	Sensitivity (%) [95% CI]	Specificity (%) [95% CI]	PPV (%) [95% CI]	NPV (%) [95% CI]
Model 1							
Using Saehan dynamometer by Spexoto et al. (2022)	Using Gripwise dynamometer by Villain et al. (2023)			100 (100-100)	31.69 (24.24-39.14)	7.62 (3.37-11.86)	100 (100-100)
Dynapenic, n	45	0	45				
Non-dynapenic, n	97	7	104				
Total, n	142	7	149				
Model 2							
Using Saehan by Spexoto et al. (2022)	Using Gripwise by Spexoto et al. (2022)			55.77 (47.79-63.74)	97.78 (95.41-100.14)	98.31 (96.23-100.38)	48.89 (40.86-56.92)
Dynapenic, n	44	46	90				
Non-dynapenic, n	1	58	59				
Total, n	45	104	149				
Model 3							
Using Saehan by Cruz-Jentoft et al. (2019)	Using Gripwise by Cruz-Jentoft et al. (2019)			16.67 (10.68-22.65)	99.20 (97.77–100.63)	80.00 (73.58-86.42)	86.11 (80.56-91.66)
Dynapenic, n	124	20	144				
Non-dynapenic, n	1	4	5				
Total, n	125	24	149				

Note: PPV, positive predictive value; NPV, negative predictive; 95% CI, 95% Confidence Interval; Model 1 = Comparison of low strength classification by Spexoto et al. (2022) (< 23 kgf for women and <36 kgf for men) using the analogic Saehan dynamometer with low strength classification by Villain et al. (2023) (< 12 kgf for women and <22 kgf for men) using the Gripwise dynamometer; Model 2 = Comparison of low strength classification by Spexoto et al. (2022) (< 23 kgf for women and <36 kgf for men) using both the analogic and Gripwise dynamometers. Model 3 = Comparison of low strength classification by Cruz-Jentoft et al. (2019) (< 16 kgf for women and <27 kgf for men) using both the Saehan and Gripwise dynamometers.

The level of agreement in model one was 65.1% (*κ* = 0.14); for model 2, the agreement was 68.5% (*κ* = 0.42); and for model 3, the agreement was 85.9% (*κ* = 0.23). Therefore, all kappa statistics were considered to reflect minimal or weak levels of agreement.

Given the need to evaluate the agreement of the use of the dynamometer in a certain age group, we chose to investigate whether these people were the most poorly classified, and this was done through additional analyzes. The group of individuals aged 80 or older was selected to assess the concordance of classifications according to the models within this age group. It is important to note that this subgroup consists of only 11 individuals. However, the results show that Model one incorrectly classified four individuals as dynapenic. Model 2, on the other hand, incorrectly classified only two individuals as dynapenic. Model three misclassified five individuals as dynapenic. Overall, although the subgroup is quite small, it is evident that Model two performed better in this age group.

## Discussion

This study assessed the reliability of the Gripwise digital dynamometer for measuring handgrip strength and compared it with the Saehan analogic dynamometer. The results, based on the intraclass correlation coefficient (ICC), indicated excellent reliability for both devices (ICC >0.90), which is consistent with findings from previous studies. Villain et al. (2023) compared Gripwise to Jamar hydraulic in a sample of 348 hospitalized older patients, observing average ICC values of 0.93 and 0.94, respectively, both classified as excellent (Villain et al., 2023).

In addition, a previous study using the BodyGrip dynamometer, a precursor of Gripwise, compared it with Jamar hydraulic in the assessment of handgrip strength. The correlation between handgrip strength measurements for the non-dominant hand, using both devices (BodyGrip with curved and straight handles), presented ICC values ranging from 0.93 to 0.95, indicating excellent reliability (Guerra et al., 2017).

Regarding Saehan equipment, its validity was compared to the gold standard Jamar hydraulic in a sample of 100 healthy individuals, finding intra-rater and inter-device validity ranging from 0.97 to 0.98, values considered excellent, concluding that data collected with Jamar are equivalent to those obtained with Saehan (Reis and Arantes, 2011). Another study examined the intra- and inter-rater reliability of the handheld dynamometer for measuring strength in 23 muscle groups in people with mild Parkinson’s disease (Bloom et al., 2023). The results showed that handgrip strength, measured with the Saehan (Model SH5003; Saehan Corporation, Masan), exhibited excellent reliability across all analyses when compared to the DIGI-II dynamometer (Model 01,163, Lafayette Instrument Inc., Indiana) (Bloom et al., 2023).

In our study, despite excellent reliability, there was a significant difference in the average values between the dynamometers, both in the three individual measurements (approximately four kgf) and in the maximum value obtained (a difference of 3.5 kgf), which had a considerable impact on the classification of dynapenia. This result supports the findings of Villain et al. (2023), who also compared Jamar hydraulic and Gripwise and observed significantly lower measurements compared to the gold standard (around three to four kgf). The lower readings were found for both the average values and the three measurements, regardless of dominant or non-dominant hand, measurement order, and participant position (sitting or lying down) (Villain et al., 2023). We raised the hypothesis that this difference may be due to the ergonomics and physical characteristics of the device itself, such as its weight. Gripwise may report lower handgrip strength values due to differences in ergonomics (dimensions: Saehan – 29 cm × 20 cm × 11 cm; Gripwise – 15 cm × 4.4 cm × 3.5 cm) and weight (Saehan – 1.4 kg; Gripwise – 290 g), as its smoother shape and lighter design provide less stability and adaptability compared to the Saehan dynamometer, despite both devices following the same data collection protocol. Another study presented similar results and explained that the lower values are likely related to the different handle shape, which induces differences in finger muscle activation. In this context, the straight shape of the Gripwise seems to be a problematic factor (Villain et al., 2023).

The analysis of handgrip strength values with different cutoff points, both those established in the literature and the specific cutoff point for Gripwise, showed that the model proposed by Spexoto et al. (2022) had the best performance in the classification of dynapenia. The results regarding this superior performance is because Spexoto’s cutoff point was formulated based on functionality, rather than the traditional method of statistical distribution curves. This cutoff point was developed considering different muscle strength values and walking speed, aiming to assess the risk of mortality in older adults, using a representative sample of 6,182 participants (Spexoto et al., 2022).

Based on our analyses and the verification of dynapenia classification, we suggest that existing cutoff points may need to be adjusted, considering differences between devices and the behavior of models in older age groups. In our study, for instance, the subgroup of individuals aged 80 years or more showed a higher classification error, being better assessed by Model 2, as previously noted, the model demonstrated the fewest errors in classifying individuals as dynapenic.

Another relevant aspect relates to the Kappa indices, which reflected weak levels of agreement (*κ* between 0.14 and 0.42). These results contrast with the study by Villain et al. (2023), which found strong agreement values for both sexes (*κ* = 0.80 for women and *κ* = 0.90 for men) (Karagiannopoulos et al., 2022). This discrepancy in the Kappa indices raises questions about the applicability of the models, suggesting that further investigations are necessary to improve the consistency of dynapenia classifications and to establish new cutoff points.

Regarding study limitations, the small sample size, a sample predominantly composed of women, should be noted, as well as the subgroup of individuals aged 80 or more, which contained only 11 participants. Another probable limitation is the comparison conducted with a dynamometer other than the Jamar. Despite these limitations, the study makes a significant contribution by applying new technology for handgrip strength assessment in older adults and also highlighting the need to be aware of these differences when choosing a cutoff value to classify dynapenia. The sample includes both men and women, encompassing healthy individuals with different levels of physical activity, which adds complexity to the analysis, allowing exploration of the potential benefits and limitations of using this emerging technology.

## Conclusion

In conclusion, this study evaluated the reliability of the Gripwise digital dynamometer in measuring handgrip strength in older Brazilian adults concerning Saehan analogical equipment, demonstrating excellent agreement for both devices (Gripwise and Saehan) with ICC values above 0.90. Significant differences in mean values between Gripwise and Saehan highlight the need for caution in equipment selection for clinical practice. Gripwise identified more cases of dynapenia, which could enable earlier detection of muscle weakness and quicker intervention. However, this may also lead to an overestimation of dynapenia prevalence if the values do not accurately reflect true muscle strength, potentially resulting in unnecessary interventions. Thus, adjusting the cutoff points for Gripwise, especially for older populations, is crucial.

## Data Availability

The original contributions presented in the study are included in the article/supplementary material, further inquiries can be directed to the corresponding author.
